# Access to and Use of Mobile Phone by Postpartum, Married Women in Punjab, India: Secondary Analysis of mHealth Intervention Pilot Data

**DOI:** 10.2196/34852

**Published:** 2022-05-12

**Authors:** Ruchita S Pendse, Alison M El Ayadi, Preetika Sharma, Alka Ahuja, Darshan Hosapatna Basavarajappa, Mona Duggal, Ankita Kankaria, Pushpendra Singh, Vijay Kumar, Rashmi Bagga, Nadia G Diamond-Smith

**Affiliations:** 1 Stanford University School of Medicine Stanford, CA United States; 2 Department of Epidemiology and Biostatistics University of California San Francisco San Francisco, CA United States; 3 Department of Obstetrics, Gynecology, and Reproductive Sciences University of California San Francisco San Francisco, CA United States; 4 Post-Graduate Institute of Medical Education and Research Chandigarh India; 5 Department of Obstetrics and Gynaecology Post-Graduate Institute of Medical Education and Research Chandigarh India; 6 Department of Community and Family Medicine All India Institute of Medical Sciences Bathinda India; 7 Department of Computer Science and Engineering Indraprastha Institute of Information Technology Delhi India; 8 Survival for Women and Children Foundation Panchkula India

**Keywords:** pregnancy, mothers, postpartum period, postnatal care, mobile phone use, mHealth, mobile health, digital health, telemedicine, health education, sex factors, gender, India, South Asia

## Abstract

**Background:**

As mobile phone uptake in India continues to grow, there is also continued interest in mobile platform–based interventions for health education. There is a significant gender gap in mobile phone access—women’s access to mobile phones is constrained by economic and social barriers. Pregnancy and postpartum care is one of many targets for mobile health (mHealth) interventions that particularly rely upon women’s access to and facility with mobile phone use.

**Objective:**

We aimed to describe the dynamics and patterns of married pregnant and postpartum women’s mobile phone access and use (among both phone owners and nonowners) who participated in an mHealth postpartum care intervention and to identify potential barriers to their participation in mobile platform–based interventions.

**Methods:**

A secondary analysis was performed on mixed methods data obtained for a pilot mHealth intervention for postpartum care of mothers in rural Punjab from July 2020 to February 2021. Two formative sources included exploratory in-depth interviews among postpartum women (n=20; 1-3 months postpartum) and quantitative maternal health survey among women who were pregnant or who had recently given birth (n=102). We also utilized mixed methods intervention assessment data from early postpartum women who participated in the pilot intervention (n=29), including intervention moderator perspectives. Qualitative and quantitative analyses were performed, and pertinent findings were grouped thematically.

**Results:**

The majority of women owned a phone (maternal health survey: 75/102, 74%; demographic survey: 17/29, 59%), though approximately half (53/102, 52%) still reported sharing phones with other family members. Sharing a phone with female family members typically allowed for better access than sharing with male family members. Some households had strict preferences against daughters-in-law having phones, or otherwise significantly restricted women’s phone access. Others reported concerns about phone use–related health hazards for mother and infant during the pregnancy or postpartum period.

**Conclusions:**

These findings suggest nuance regarding what is meant by women’s phone ownership and access—there were numerous additional constraints on women’s use of phones, particularly during pregnancy and the postpartum period. Future research and mHealth interventions should probe these domains to better understand the dynamics governing women’s access, use, and fluency with mobile phones to optimally design mHealth interventions.

## Introduction

Mobile phone use is growing dramatically globally, including in India. India’s subscriber base for mobile devices in 2015 was 867.8 million (64.8% of the country’s population) and was slated to rise exponentially by 2019 due to a 4-fold increase in mobile commerce sales. [1]. In the Indian states of Punjab and Haryana alone, over 70% of internet users access wireless networks through mobile devices [2]. Smartphone penetration was predicted to rise to 800 million users before the end of 2019 [1]. Younger people are even more likely to use mobile phones; over 25% of retail transactions in India are web-based using mobile phones, with the highest reported use being in the age group of 18- to 34-year-old adults [3].

Mobile phone use has expanded to health care as well in India, with more people opting for telehealth provider interactions [3]. Thus, there is increased possibility for reaching people, including women, with health care information and support using mobile phones through mobile health (mHealth) interventions. mHealth approaches encompass interventions that use some type of mobile phone–based technology to provide health information or services. There have been a plethora of mHealth interventions globally that have targeted pregnancy, maternal, child, and reproductive health using approaches that include text messages, hotlines, and communication platforms that connect women to community health workers, doctors, or each other [4]. mHealth interventions have been shown to successfully improve dietary intake in pregnant women [5] and health service utilization during pregnancy, delivery, the postpartum period, and for child health [4,6]. mHealth interventions have particular potential value for women in the postpartum period in India where postpartum visits are below recommendations [7]. Significant logistical barriers prevent mothers from physically attending postnatal care appointments at facilities or other locations that may be far from their homes, particularly in India [8]. Common logistical challenges, such as difficulty obtaining transportation and with scheduling, are exacerbated in India by rural geographic distances, cultural and linguistic barriers to care, women’s practice of postnatal seclusion, and generally low levels of mobility for married women [9-11]. Further intergenerational and gender-based hierarchical roles structure decision-making in Indian households, particularly for couples living in extended-family households, with decision-making largely outside of the hands of mothers [12], especially those who are young and newly married. Despite women’s physical mobility limitations in this setting, there is broad access to mobile telephones in India with 88% of households nationally owning a mobile phone [13].

Despite high household phone ownership, substantial gender disparities in phone ownership and use exist in India. In fact, South Asia has the largest gender gap in phone ownership of any region globally [14]. A 2019 report [15] highlighted some of these gaps, finding that 75% of men and only 51% of women owned mobile phones in India. This gap is even more pronounced for smartphone ownership, with 37% of men owning smartphones in 2019 compared to only 14% of Indian women [16]. Men are more likely not only to own a phone, but also to make calls, receive calls and text messages, and access the internet [15]. Women phone users may be expected to spend small amounts of time on the phone and to do so mostly within their homes. Unequal gender norms and conceptions of women’s roles in the household impact phone use as well, as phone use may be considered to be associated with risks to women’s purity or reputation, and can also be seen to be in conflict with women’s household responsibilities. Other research has suggested that women are perceived to not need a phone or that their use should be censored [17]. The gender gap in phone use is highest in adolescence and early marriage—when fears about women’s reputations are most pronounced [15].

While there is this large gender gap in ownership of phones, Indian women often do report access to a shared phone within the household [15]. This access to a shared phone, alongside the growing population of women phone owners, has been the basis for numerous mHealth interventions targeting women, including those pertaining to maternal health [4-6,18-25]. However, because men are often the gatekeepers to these shared phones, women often have less access to these phones, either indirectly, because the men take the phones with them and are apart from women while at work, or directly, because men choose to limit how much women use the phone [15,26]. While there is increasing information about the gender gap in phone ownership and its impact on access to phones, we know little about how gender norms and inequalities around mobile phones specifically impact women’s ability to participate in mHealth interventions, including those targeting women themselves.

We aimed to explore how gender, mobile phone access, and mobile phone use patterns intersected to structure women’s participation in an mHealth intervention to improve access to high quality postpartum care and social support for women in rural India.

## Methods

We developed an intervention to improve access to postpartum care and social support for women in rural India that included group calls over the phone and additional interaction and content over WhatsApp. We followed a human centered design process; initial stages included a formative mixed methods phase and a pilot [27] of the intervention, which also included mixed methods evaluation.

We conducted secondary analysis of these data—exploratory in-depth interviews, a maternal health survey, and intervention assessments (Table 1). We developed interview guides and survey questions and pilot tested the tools among respondents from the same population in which we aimed to collect data; respondent feedback was used to improve the interview guides and survey questions. For the quantitative survey, where possible, validated measures were used, such as items from the National Family Health Survey of India [7]; however, we ultimately developed most items, since there were no standardized validated measures for phone use patterns specific to this population of women.

Participants for the exploratory in-depth interviews and for the maternal health survey were recruited from antenatal and birth registries in the study area and over the phone by study team members. In-depth interviews were scheduled and conducted in person (usually lasting 45 minutes and conducted at the participant’s home). The interview guide included questions on conception, women’s experiences across the continuum of perinatal care (ie, antenatal, childbirth, and postnatal), neonatal experiences, and women’s acceptability of and preferences regarding mobile phone–based interventions. Maternal health survey participants were recruited over the phone; the survey was also administered over the phone and included questions regarding mobile phone access and use and regarding recent pregnancy and childbirth care experiences.

Participants for the mHealth postpartum information and social support pilot intervention were recruited from birth registries. Inclusion criteria were having their residence in study area, having given birth within the prior 2 weeks, being <40 years of age, and having a live neonate >1500 grams. Exclusion criteria were complications for the mother during or after childbirth warranting hospital stay and continued medical care at the facility, stillbirth, twins, significant birth defects, inability to provide informed consent, and lack of phone access if they were unwilling to accept a phone from the study team. A study researcher explained the study procedures in detail over the phone, including the risks and benefits of participation. Participants provided verbal consent. Where requested, assent was obtained from the husband or another family member (in alignment with local norms). Participants were sequentially enrolled into 3 groups (total: n=29 women; group 1: n=7, group 2: n=10; group 3: n=12) based on their child’s birth date. They completed a short demographic questionnaire at enrollment that included questions about mobile phone ownership (none, individually owned, shared with another household member), mobile phone type (smart versus feature phone), and willingness to accept a mobile phone from the study team if they did not have their own mobile phones. We conducted a brief phone-based survey on a weekly basis regarding participants’ experience with the intervention, what they liked, what they did not like, and any challenges that they experienced. In-depth interviews were conducted with a subset (n=15) of participants after the 6-week intervention had been completed. Research team members moderating group sessions tracked what worked and what did not work after each group call in a structured text format.

Quantitative data were analyzed descriptively (i.e. frequencies and proportions, by data collection source). For qualitative data, we followed a 2-stage systematic process: (1) deductive and emergent coding, and (2) thematic analysis (the different dimensions and commonalities, their distribution across sociodemographic variables, and the patterns and linkages between themes). Qualitative data were coded in Dedoose cloud-based software. Findings and interpretations were discussed by the full research team.

**Table 1:** Mixed methods data sources combined for phone use analysis.

Source	n	Participants	Time frame
Exploratory in-depth interviews	20	1-3 months postpartum	July to December 2020
Maternal health survey	102	Pregnant or early postpartum	January to February 2021
Demographic survey	29	Early postpartum women participating in our pilot intervention	November to December 2020
Weekly check-in surveys	29	Early postpartum women participating in our pilot intervention	December 2020 to January 2021
Postintervention in-depth interviews	15	Early postpartum women participating in our pilot intervention	January 2021

## Results

### Phone Owners

The majority of women reported having a phone (maternal health survey: 75/102, 74%; demographic survey: 17/29, 59%). Most had access to a smartphone (87/102, 85% in maternal health survey and 29/29, 100% in demographic survey); in some cases, women themselves owned a feature phone but had access to a household smart phone. However, even when women reported owning a phone, 53 of the 102 women (52%) in the maternal health survey reported they still had to share it with others, typically with other women in the household (such as sister-in-law or mother-in-law). When women reported they did not own a phone but had shared access to one, the owner was most commonly their husband.

The maternal health survey found that almost all phone owners use their phone for voice calls (68/75, 91%) and WhatsApp (57/75, 76%) (Table 2). Only approximately 1 in 5 used it for internet (15/75, 20%) or for videos (14/75, 19%), and less than 10% used SMS or texting (6/75, 8%).

**Table 2:** Phone use by maternal health survey respondents from northern India.

Type of use	Respondents with phones (n=75), n (%)
Voice calls	68 (91)
SMS or text	6 (8)
WhatsApp	57 (76)
Facebook	21 (28)
Internet	15 (20)
Watching videos	14 (19)
Other	17 (23)

### Nonphone Owners

Of the 102 respondents to our maternal health survey, 27 (26%) women did not have their own phone. However, even these nonowners had household access to phones, with 11 (41%) reporting daily use ranging from 15 to 60 minutes per day, 5 (19%) reporting only once weekly use, and another 11 (41%) reporting less than weekly use.

Women who participated in the in-depth interviews reported sharing access to phones owned by husbands, sisters-in-law, mothers-in-law, and other family members. They generally reported a single phone (as the shared phone) in the household, though some shared access to multiple household phones as the need arose. Among those who shared a phone, they often reported limited use, in some cases only to receive or make calls to their family. One respondent explained that she could use her mother-in-law's phone just to make a call but had to give it back right away and that she did not use messaging or any other phone features.

### Husband’s Ownership of Phone

For women whose mobile phone access was through their husband, the husband’s work outside the home and his own phone use often precluded women’s phone use, especially during their husband’s working hours. One woman described the various approaches she used to try to participate in the intervention and the barriers she had faced:

I have only attended the first call. I attended that call on my neighbor’s phone. Now they go for work. This is my husband's number. He is also always at work.

Women’s travels to their natal homes, which is common in pregnancy and the postpartum period, limited their access to husbands’ phones. Even in a woman’s natal home, if only men had phones, the woman’s access to their brother or father’s phone would be similarly limited by the hours the male relative was in the home. When women shared phones with another female household member (sister-in-law or mother-in-law) they were more likely to have better access throughout the day.

Communications intended for women who were not the primary owners of a device were sometimes subject to censoring or control by the primary owner. Some women reported that their husband listened to the information and passed it along to her. Other husbands sometimes did not relay messages or contact attempts to their wife, or acted as a barrier to direct contact by conveying messages rather than offering alternative contact numbers for their wives. Additionally, in some cases, husbands left the messaging groups intended to provide information or support for their wife.

Women noted that phone access was more restricted in their husbands’ homes than in their natal homes (before they were married). One woman described how

...before marriage I had my personal phone and used it, but after marriage I have not kept any personal phone.

When asked if she felt she still needed a phone, she replied,

no, not too much. There is always someone or the other at home so I don’t feel the need of it.

### Family Attitudes Against Women’s Phone Ownership

Some women said that their family explicitly stated that they would not allow women’s phone ownership. One woman explained that her

in-laws will not agree (with my phone ownership). They do not like if we keep a phone.

Another woman initially was amenable to phone ownership, then consulted her husband and declined, stating that

it is looked down upon if women in the family (keep a phone). No women in the family, including the mother and sisters-in-law, keep a phone.

Notably, several women who were unsure about the acceptability of phone ownership explained that they would have to ask for permission from their husband or in-laws to accept a phone or to join a WhatsApp group. Even some women who were phone owners noted they would need to obtain permission to participate in a WhatsApp group.

### Beliefs About Phones and Maternal and Child Health

Some mothers articulated beliefs about mobile phones and other electronics being harmful to the health and development of a fetus or young child. One woman explained that

doctors do not permit use of mobile phones during pregnancy and near a child. I don’t have a smartphone but my husband has one, so when he is home I use it a little bit to watch videos... [My husband] permitted me only one hour to use the phone, not much, because its rays are harmful for the development of the child’s heart.

When asked which doctor had told her this, she said it was a doctor at a dispensary.

Another mother shared that

...[my in-laws] would say that during [pregnancy I should] keep the phone away from me as it affects the baby. Now I keep the ringtone off since she was born. Before that also I would keep vibration off and would keep it away.

Another woman reported that she had not used a mobile phone during pregnancy at all, and that

[my husband] also stopped T.V. and all for me saying that you shouldn't use anything like this that will affect the baby. Even after she was born I haven’t seen that much. Now that it has been 3-4 months, I have started checking phone a little bit. He didn’t allow me to watch even a little bit and said our safety is in our hands. Tomorrow if any problem arises then also it will affect us. He said that if I will see phone then I will get addicted to it and it will become the habit of the baby. I didn’t use the phone for 9 months.

This concern about the child getting habituated to a phone was shared by another mother as well, who noted that

[my husband] was saying to me also to get a phone but I am not getting one because of the baby. You know that it spoils the habits of the children. Right now my brother-in-law shows [my child] the phone and he keeps on staring at it. I feel that he will get used to it and will have this habit.

### Women’s Household Responsibilities

Women reported household responsibilities as a significant and frequent barrier to phone use, which precluded availability even during scheduled weekly times. One woman shared

there was lot of work at home. My mother-in-law is sick already and with two kids, I barely get time for anything.

Participants in our intervention, who had recently given birth, reported childcare and household responsibilities as being substantial barriers to their participation.

### Concern for Mobile Fraud

Some women reported that they were concerned that they would receive calls or messages on mobile devices from unknown numbers due to experiences with mobile fraud personally or having heard about people seeking financial information over the phone. One woman mentioned that

actually I may have missed the first 1-2 calls as my sister-in-law was hesitant and she told me so. But then I thought let’s pick up and see what happens. Then we got confident that it’s not a fraud call.

## Discussion

### General Context

We focus on the implications of these findings for future researchers and implementors designing mobile platform-based interventions for women in South Asia. Women’s access to and ownership of mobile phones in India continues to increase, promoting optimism about the associated benefits to women’s agency, economic empowerment, access to education, access to health resources, and opportunities for mobile platform–based interventions for health alongside other fields [15,22]. However, there have recently been some calls for caution in designing mHealth and other mobile platform–based interventions, given awareness of gender inequities in mobile phone use and access, and concern that these interventions may have potential to exacerbate these inequalities [21,22,28].

### Principal Findings

Consistent with previous studies [15,17,28], in our study, we found that women reporting access to but not ownership of mobile phones may have very limited use of the device. Women whose phone access occurred through their husbands may lack access to a phone during the day when husbands are out of the house for work, thus they cannot be reached and cannot participate in mHealth interventions during that time. The same physical and logistical barriers that have been found to limit women’s ability to participate in phone surveys can also hinder women’s ability to participate in mHealth interventions [15].

Existing mHealth literature tends to dichotomize women’s phone access based on phone ownership, where ownership may be assumed to portend greater autonomy and access than simply having access to a shared phone. However, we found there may also be significant constraints to women’s use of their own phones, particularly if their phone they own is still shared. Of note, when women reported ownership of a phone that was shared, they more often shared with other female relatives rather than with male relatives. When phones were shared among women, they tended to maintain better access to the phone as compared to sharing with their husband, both indirectly because men left for work during the day, and directly because of some husbands’ attempts to restrict or control women’s phone access. These findings suggest that future researchers should probe all women, including phone owners, to determine whether they share a mobile device and should understand who the phone is being shared with in order to understand a fuller picture of women’s mobile access.

Our findings also highlight that gender norms that have previously been identified as having an impact on women’s phone ownership and use are also barriers to their participation in mHealth interventions. As in previous studies [15,17,28], our participants described challenges due to household responsibilities as well as perceptions about if, when, and how much women should be using phones. In particular, we encountered specific concerns about phone use during pregnancy and in the postpartum period, and its impact on the health and habits of the mother and infant. While these beliefs did not appear to be widespread, they had the potential to dramatically alter a woman’s phone access during pregnancy and the postpartum period, with one woman reporting almost complete avoidance of technology for the duration of her pregnancy and 3 months postpartum. We also identified concerns over potential fraud, as others have found [15], despite the fact that our initial recruitment and interaction for the intervention was through trusted and known institutions. This suggests that fears and myths about fraud over the phone might be a substantial barrier. Our findings extend the literature by describing how women in pregnancy and in the postpartum period may be especially limited in their ability to engage with mobile phones due to beliefs about the health and safety of women using phones while pregnant or infants being close to mobile phones. These types of beliefs act as substantial barriers and must be considered and explored further. Of note, some mHealth intervention designs include the option of providing a mobile phone or smartphone to participants. While this may address economic barriers to a participant’s phone ownership, it does not address, and in fact may grate against, sociocultural barriers to women’s phone use [21].

We also observed surprising phone use patterns in our survey of pregnant and postpartum women. Almost half (11/27, 41%) of nonowners who reported shared access to a phone actually used a phone less than weekly. We are not aware of previous recent studies characterizing frequency of phone use among women reporting shared access, and a recent meta-analysis of literature on women’s mobile phone access similarly did not address frequency of use [17]. This is an important future avenue to explore in order to better characterize the diversity of experiences represented by Indian women who report shared phone access. Even smartphone owners report relatively low utilization of internet services other than WhatsApp, consistent with prior findings that the gender gap widens with sophistication of task (eg, taking calls vs using WhatsApp vs surfing the web) [15,17,28]. This has important implications for the development of maternal mHealth interventions that may rely on some level of fluency with app interfaces and other more sophisticated tasks.

### Limitations

This study had several limitations. First, data came from different data sources, collected from women who were pregnant or had recently given birth, including both participants in our intervention as well as nonparticipants. The data were not collected synchronously, though they were all collected within 6 months of each other. We also included data collected from participants after completion of the intervention pilot. These differences in participant populations and time points of data collection may limit the generalizability of our findings. Additionally, the generalizability of our findings is constrained by the study population being only recruited in rural Punjab, and our observed use patterns and beliefs may not be applicable to women living in urban areas or other regions of India.

Of note, the question addressed by this paper—describing women’s mobile phone use patterns—was not one of our initial research questions. The themes surrounding women’s mobile phone beliefs and use emerged from data being collected for the intervention design and evaluation. While this limits both the specificity and the breadth of our findings, we present only what women organically shared with us in the process of discussing the themes that we probed, with some additional quantitative context provided by our surveys. We are also accordingly constrained by collecting data only from women, whereas truly exploring this question of gender norms surrounding women’s mobile phone use would benefit from the perspectives of other family members. Future exploration of this space can build on our findings by incorporating these familial perspectives as well as additional measures of empowerment to better characterize women’s use of mobile phones especially for mHealth interventions during and after pregnancy.

### Conclusion

As mobile technology access continues to expand in India and other countries, mobile platform–based interventions remain a promising avenue for education and information dissemination, particularly for health-related topics that carry significant potential to improve individual and population health and well-being, such as maternal and reproductive health. Pregnancy and postpartum care, in particular, have been targeted due to the high risks to mother and infant during this time, and significant room for improvement in meeting care milestones set by the Indian government and World Health Organization [7,29]. Our findings suggest that additional attention should be paid to women’s phone access beyond reports of ownership or access to phones, as there may be considerable variation and constraints in the duration and timing of women’s access, beliefs around mobile phone health hazards, and women’s degree of facility with mobile phone functions. A better understanding of these nuanced factors will facilitate maternal mHealth intervention design and success, and may have implications for other health topics, although more research is needed.

## Abbreviations

mHealth: mobile health
